# The Chromatin Environment Around Interneuron Genes in Oligodendrocyte Precursor Cells and Their Potential for Interneuron Reprograming

**DOI:** 10.3389/fnins.2019.00829

**Published:** 2019-08-08

**Authors:** Linda L. Boshans, Daniel C. Factor, Vijender Singh, Jia Liu, Chuntao Zhao, Ion Mandoiu, Q. Richard Lu, Patrizia Casaccia, Paul J. Tesar, Akiko Nishiyama

**Affiliations:** ^1^Department of Physiology and Neurobiology, University of Connecticut, Storrs, CT, United States; ^2^Connecticut Institute for Brain and Cognitive Sciences, University of Connecticut, Storrs, CT, United States; ^3^Department of Genetics and Genome Sciences, School of Medicine, Case Western Reserve University, Cleveland, OH, United States; ^4^Computational Biology Core, University of Connecticut, Storrs, CT, United States; ^5^Advanced Science Research Center at the Graduate Center, Neuroscience Initiative, The City University of New York, New York, NY, United States; ^6^Department of Pediatrics, University of Cincinnati, Cincinnati, OH, United States; ^7^Department of Computer Science and Engineering, University of Connecticut, Storrs, CT, United States; ^8^Institute for Systems Genomics, University of Connecticut, Storrs, CT, United States

**Keywords:** NG2, oligodendrocyte, inhibitory neuron, reprograming, chromatin, histone post-translational modification

## Abstract

Oligodendrocyte precursor cells (OPCs), also known as NG2 glia, arise from neural progenitor cells in the embryonic ganglionic eminences that also generate inhibitory neurons. They are ubiquitously distributed in the central nervous system, remain proliferative through life, and generate oligodendrocytes in both gray and white matter. OPCs exhibit some lineage plasticity, and attempts have been made to reprogram them into neurons, with varying degrees of success. However, little is known about how epigenetic mechanisms affect the ability of OPCs to undergo fate switch and whether OPCs have a unique chromatin environment around neuronal genes that might contribute to their lineage plasticity. Our bioinformatic analysis of histone posttranslational modifications at interneuron genes in OPCs revealed that OPCs had significantly fewer bivalent and repressive histone marks at interneuron genes compared to astrocytes or fibroblasts. Conversely, OPCs had a greater degree of deposition of active histone modifications at bivalently marked interneuron genes than other cell types, and this was correlated with higher expression levels of these genes in OPCs. Furthermore, a significantly higher proportion of interneuron genes in OPCs than in other cell types lacked the histone posttranslational modifications examined. These genes had a moderately high level of expression, suggesting that the “no mark” interneuron genes could be in a transcriptionally “poised” or “transitional” state. Thus, our findings suggest that OPCs have a unique histone code at their interneuron genes that may obviate the need for erasure of repressive marks during their fate switch to inhibitory neurons.

## Introduction

Oligodendrocyte precursor cells (OPCs), also known as NG2 glia, NG2 cells, or polydendrocytes, are ubiquitously present throughout the central nervous system and comprise 2-9% of total cells (Dawson et al., 2003). They represent a fourth major population of glial cells endowed with proliferative and self-renewing ability throughout life. Their most well known function is to generate oligodendrocytes in the developing and mature central nervous system, but they also exhibit some degree of lineage plasticity, as briefly reviewed below. The term NG2 glia has been used in the context where the properties of these cells other than their role as oligodendrocyte-producing cells is discussed. However, there has been no evidence that a subpopulation of OPCs generates oligodendrocytes, while other distinct subpopulations receive inputs from neurons (Bergles et al., 2000) or generate astrocytes (Zhu et al., 2008, 2011). On the contrary, accumulating evidence supports the notion that neuronal inputs onto OPCs affect the dynamics of oligodendrocyte lineage cells (Gibson et al., 2014; Hill et al., 2014). Thus, the consensus in the field is that NG2 glia are equated with OPCs and represent cells that have the potential to generate oligodendrocytes but have other functions as well (Nishiyama et al., 2016). OPCs are identified by the expression of NG2 and platelet-derived growth factor receptor alpha (PDGFRα) (Nishiyama et al., 1996, 2016). Neither protein is exclusively present in OPCs. NG2 is also expressed by vascular pericytes and a subpopulation of macrophages that enter the CNS (Stallcup et al., 2016) though not on resting ramified microglia (Nishiyama et al., 1997). *Pdgfra* transcript is also present at a low level in neurons and other unidentified cell types, though it is >60-fold more abundant in OPCs (Vignais et al., 1995; Zhang et al., 2014). Thus, it has become the convention to identify OPCs by the combinatorial expression of the oligodendrocyte transcription factor Olig2 (see below) and one of the two cell surface antigens, NG2 or PDGFRα.

### Development of OPCs and Their Close Relation to Interneurons

During mid-embryonic development, OPCs arise in discrete domains in the ventral germinal zones, and this process is dependent on the basic helix-loop-helix (bHLH) transcription factor Olig2 (Lu et al., 2002; Takebayashi et al., 2002; Zhou and Anderson, 2002). Olig2 induces Sox10, a member of the SoxE family of high mobility group (HMG) box-containing transcription factors. The onset of Sox10 expression marks the commitment to the oligodendrocyte lineage (Kuhlbrodt et al., 1998; Kuspert et al., 2011). This is shortly followed by their emigration from the germinal zone and onset of expression of NG2 and PDGFRα (Nishiyama et al., 2016; Weider and Wegner, 2017). In the forebrain, a subset of OPCs is generated from ventral neural progenitor cells (NPCs) in the ganglionic eminences, which also give rise to interneurons (Spassky et al., 1998; Nery et al., 2001; Kessaris et al., 2006; Miyoshi et al., 2007). Ventral NPCs express the pro-interneuron homeodomain transcription factors Dlx1 and 2, and when Dlx1/2 expression is sustained, these cells become GABAergic interneurons. A subpopulation of these NPCs down-regulate Dlx1/2 and up-regulate Olig1/2. Cross-repression of Dlx1/2 and Olig1/2 plays an important role in the determination of interneuron and oligodendrocyte cell fates (Petryniak et al., 2007; Silbereis et al., 2014). Once specified, OPCs do not revert to a neuronal fate under physiological conditions, and they either self-renew or differentiate into oligodendrocytes (see below for more discussion on neuronal fate of OPCs) (Nishiyama et al., 2009, 2016).

Additional OPCs that arise from the dorsal germinal zones of the spinal cord expand and migrate ventrally to become intermingled with the first cohorts of OPCs that arise ventrally (Cai et al., 2005; Fogarty et al., 2005; Vallstedt et al., 2005). In the forebrain, the dorsal progenitors arise in the ventricular zone of the dorsal pallium characterized by the expression of the homeodomain transcription factor Emx1 (Kessaris et al., 2006; Winkler et al., 2018). Both populations appear to be PDGF and PDGFRα-dependent (Calver et al., 1998; Fruttiger et al., 1999). In addition, there is a small subpopulation of OPCs that appears to arise perinatally around the lateral ventricles as well as in the hindbrain in the absence of PDGF signaling (Timsit et al., 1995; Spassky et al., 1998; Zheng et al., 2018), and in the forebrain this population rapidly generates oligodendrocytes (Zheng et al., 2018). However, the exact origin of this subpopulation, its relationship to other oligodendrocytes, and the target axons they myelinate remain unclear.

### OPC-Astrocyte Fate Plasticity

Oligodendrocyte precursor cells exhibit some degree of lineage plasticity under developmental and pathological conditions. For example, some OPCs in the prenatal ventral gray matter downregulate oligodendrocyte lineage genes and become protoplasmic astrocytes, contributing to as many as one-third of the local astrocyte population, while at the same time generating oligodendrocyte lineage cells in the same region (Zhu et al., 2008, 2011; Huang et al., 2014). The ability of OPCs to become astrocytes is restricted to the ventral gray matter and is never seen in white matter tracts throughout the neuraxis. OPCs also switch their fate from oligodendrocytes to protoplasmic astrocytes upon deletion of Olig2 (Zhu et al., 2012; Zuo et al., 2018). The fate switch mediated by loss of Olig2 occurs only in OPCs in the dorsal forebrain but not in the ventral gray matter and becomes less efficient with age. Deletion of histone deacetylase 3 (HDAC3) in OPCs causes downregulation of Olig2 and phenocopies Olig2 deletion (Zhang et al., 2016). Curiously, the distribution of OPCs in the postnatal brain that are converted into functional protoplasmic astrocytes by Olig2 deletion coincides with the distribution of OPCs that arise in the dorsal germinal zone defined by the expression the homeodomain transcription factor, Emx1 (Kessaris et al., 2006; Zhu et al., 2012; Winkler et al., 2018). The differences in the astrocyte fate and fate potential of OPCs in ventral and dorsal forebrain could arise from differences in the chromatin environment of OPCs from the two different sources.

### The Neuronal Fate of OPCs

The neuronal fate of OPCs has been highly debated. Earlier studies showed that exposure of OPCs from postnatal rat optic nerves to bone morphogenetic protein 2 (BMP2) caused them to revert to a neural stem cell-like state, upregulate Sox2, and subsequently differentiate into neuron-like cells in culture (Kondo and Raff, 2000, 2004). Sox2 belongs to the SoxB1 family of HMG box-containing transcription factors and is necessary for neural stem cell maintenance (Graham et al., 2003; Thiel, 2013). It is also used as one of the four transcription factors to induce pluripotency in somatic cells (Takahashi and Yamanaka, 2006). In OPCs in the postnatal rat optic nerve, Sox2 expression is repressed by methylation at lysine residue 9 of histone H3 (H3K9), and derepression of Sox2 is critical for their fate change to neuronal cells (Lyssiotis et al., 2007).

More recent genetic fate mapping studies suggest that it is unlikely that neurons comprise a significant physiological progeny of OPCs (Dimou et al., 2008; Rivers et al., 2008; Zhu et al., 2008, 2011; Kang et al., 2010). A few studies have detected a small number of neuronal cells in different CNS regions (Rivers et al., 2008; Guo et al., 2010; Robins et al., 2013), but the findings have not yet revealed a consistent rule regarding the location or the functional subtype of neurons that are generated from OPCs, and one cannot rule out the possibility that neurons are detected in the genetic fate mapping studies due to ectopic expression of the cre recombinase in common progenitor cells or mature neurons (Nishiyama et al., 2014; Tognatta et al., 2017). Since OPCs are unique from other CNS cell types in that they remain proliferative through adulthood, one can combine genetic fate mapping with continuous labeling with 5-ethynyl-2′-deoxyuridine (EdU), which results in EdU incorporation into >98% of OPCs in young adult mice. Under these conditions, although >96% of oligodendrocytes were also EdU+, none of the neurons previously interpreted to have originated from OPCs (Rivers et al., 2008) had incorporated EdU (Clarke et al., 2012). This further suggests that proliferating OPCs do not generate neurons under normal physiological conditions.

### Direct Neuronal Reprograming From Oligodendrocyte Lineage Cells

Since the demonstration that four transcription factors could revert differentiated somatic cells to a pluripotent state (Takahashi and Yamanaka, 2006), efforts have shifted toward achieving direct reprograming from one differentiated cell into another differentiated cell type. Direct neuronal reprograming has been achieved by transfecting fibroblasts with three transcription factors *Ascl1*, *Brn2*, and *Myt1l* (Vierbuchen et al., 2010). Attempts have been made to directly reprogram neurons from OPCs. Glutamatergic and GABAergic neurons were reported to have been generated from reactive glial cells in the injured neocortex following retroviral transduction with the proneural bHLH transcription factor *Neurod1* (Bertrand et al., 2002; Guo et al., 2014). However, the identity of the cells that were initially transduced by *Neurod1* remains uncertain. Genetic fate mapping was used to show that Sox10-expressing OPCs in the injured but not intact neocortex could be converted into neurons by retroviral delivery of Sox2 and Ascl1, another member of the bHLH family (Heinrich et al., 2014), although most of the transduced cells were functionally immature, compared to neurons reprogramed from astrocytes (Heinrich et al., 2010). While these two studies only succeeded in reprograming from OPCs in the injured cortex, another study (Torper et al., 2015) showed that neurons could be generated from OPCs in the normal adult striatum by adeno-associated viral (AAV) delivery of a combination of three transcription factors *Ascl1*, *Lmx1a*, and *Nurr1*, known to promote reprograming of fibroblasts into dopaminergic neurons (Caiazzo et al., 2011). The converted neurons were stably integrated into the circuit, and many exhibited electrical properties of mature GABAergic neurons (Pereira et al., 2017). When oligodendrocytes in adult rats were transduced with an oligodendrocyte-tropic AAV harboring microRNA against polypyrimidine tract-binding protein, some of the transduced cells differentiated into neurons (Weinberg et al., 2017). However, the mechanisms by which direct neuronal conversion from glial cells occurs have remained unclear. Elucidation of basic mechanistic principles that promote or hinder direct neuronal reprograming would facilitate the application of reprograming strategies to rectify pathological conditions in which the balance of excitation and inhibition in the neural circuit is shifted toward too much excitation, such as epilepsy.

### Transcription Factors and Chromatin Regulators in Cellular Reprograming

Somatic cell reprograming using transduction of four factors *Oct3/4*, *Sox2*, *c-Myc*, and *Klf4* resets the epigenetic state of a differentiated cell into an induced pluripotent state, with changes in DNA and histone methylation at the key transcription factors (Wernig et al., 2007). The efficiency of reprograming into a pluripotent state drastically increases when the physical barrier created by nucleosomes around the pluripotency factors is removed to create an open chromatin state that is accessible for transcription factor binding (Ehrensberger and Svejstrup, 2012). ATP-dependent chromatin remodeling enzymes and/or posttranslational modification of histones play an important role in altering the chromatin state during somatic cell reprograming to induced pluripotent state. Overexpression of the BAF complex (Brg/Brahma-associated factors), one of the four ATP-dependent chromatin remodeling complexes (Hota and Bruneau, 2016), accelerates and increases the efficiency of the generation of induced pluripotent stem cells from fibroblasts by opening the chromatin, thereby obviating the need for c-Myc in the reprograming process (Singhal et al., 2010). Moreover, histone posttranslational modifications such as methylation and acetylation serve as a “code” that is read by “histone readers” that recruit molecular complexes, leading to nucleosome reorganization and restructuring of the chromatin landscape. Inhibition of enzymes that promote chromatin condensation, such as DNA methyltransferases and histone deacetylases, can increase the efficiency of generating induced pluripotent cells (Shi and Jin, 2010). Thus, it is likely that the chromatin landscape of a differentiated cell affects the ability of the cell to undergo reprograming. However, little is known about the chromatin landscape around neuronal genes in committed glial cells that might affect their neuronal reprograming efficiency.

### Chromatin Regulators in the Oligodendrocyte Lineage

Epigenetic factors play critical roles in the oligodendrocyte lineage and have been studied primarily in the context of the regulatory mechanisms that affect terminal oligodendrocyte differentiation and myelination. Inhibiting histone deacetylases and their targets in OPCs not only compromises oligodendrocyte differentiation (Shen et al., 2005; He et al., 2007; Ye et al., 2009) but also upregulates astrocyte and neuronal genes (Liu et al., 2007). While histone methylation does not appear to have a major role in oligodendrocyte differentiation, H3K9 methylation but not H3K27 methylation increases in oligodendrocytes with age (Liu et al., 2015). In OPCs, H3K9me3 occupancy is prominent on genes involved in GABA signaling, and in mature oligodendrocytes, H3K9me3 is associated with genes involved in neuronal differentiation. H3K27me3 is also associated with genes involved in neuronal differentiation in both OPCs and oligodendrocytes.

In addition to histone posttranslational modification (histone PTM), ATP-dependent chromatin remodeling has also been implicated in the oligodendrocyte lineage. A member of the SWI/SNF complex Brg1 (Brahma-related gene 1) is associated with the regulatory region of myelin genes and forms a complex with Olig2 (Yu et al., 2013; Bischof et al., 2015; Matsumoto et al., 2016). The chromodomain-binding proteins comprise another family of ATP-dependent chromatin remodeling complex. Of these, Chd7 and Chd8 cooperatively bind to key oligodendrocyte lineage transcription factors including Olig2 and Sox10 and affect proliferation, survival, and oligodendrocyte maturation (Marie et al., 2018). Furthermore, Chd8 functions upstream of Brg1 and initiates a cascade of nucleosome remodeling events mediated by Brg1 and Chd7 (Zhao et al., 2018).

While the mechanisms that regulate chromatin landscape in OPCs are beginning to be unraveled, these studies have been conducted in the context of regulation of cellular dynamics within the oligodendrocyte lineage and have focused primarily on their function at oligodendrocyte and myelin genes. There is currently little information on how these mechanisms affect the ability of OPCs to undergo reprograming into other cell types. Chromatin immunoprecipitation sequencing (ChIP-seq) for *Ezh2*, which is the catalytic component the Polycomb Repressor Complex 2 and catalyzes histone methylation to generate H3K27me3, has revealed that *Ezh2* is enriched at many of the genes that promote neuronal fate or neuronal differentiation (Sher et al., 2012). Based on these observations and the close relationship between interneuron and oligodendrocyte development, we hypothesized that interneuron genes in OPCs are modified by histone PTMs that facilitate their reprograming into interneurons. To test this, we have systematically analyzed active, latent, bivalent, and repressive histone marks at the promoter and distal regions of interneuron genes in OPCs and compared them with those in astrocytes and fibroblasts.

## Materials and Methods

### Interneuron Gene Expression in OPCs and Other Cell Types

**FIGURE 1:**
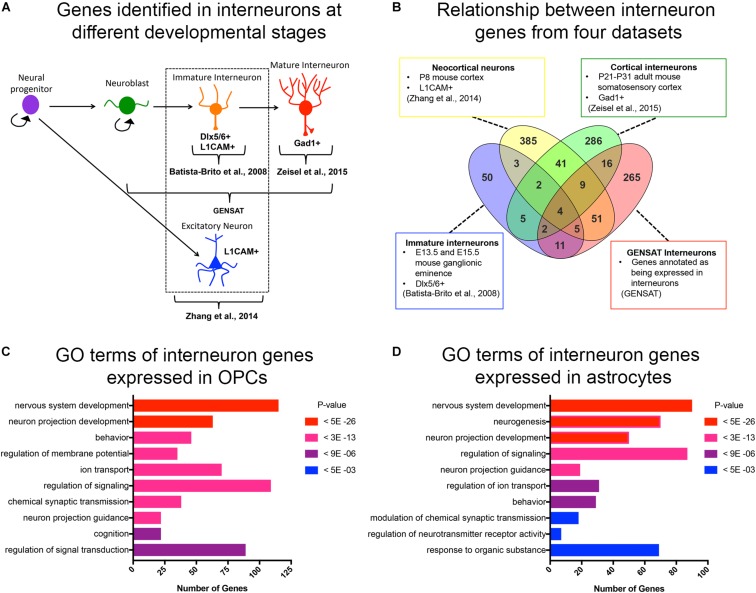
Curated list of interneuron genes. **(A)** Diagram illustrating the stages of interneuron development and the sets of interneuron genes used. Markers used to identify interneuron populations are shown. **(B)** Venn diagram displaying the number of genes obtained from each source and the degree of overlap. A total of 500 genes from P8 neocortical neurons, 82 genes from immature neurons from embryos, 365 genes from cortical interneurons from juvenile mice, and 372 genes from GENSAT annotated interneurons. **(C,D)** Bar graphs of the top 10 significant gene ontology (GO) terms (*y-*axis) of interneuron genes expressed in OPCs **(C)** and astrocytes **(D)**. The number of genes in each GO category is shown on the *x-*axis. GO categories are ordered by *p*-values. Red represents the most significant GO terms while blue represents the least significant.

To compile a list of interneuron genes, we curated genes that are important for interneuron development, function, and identity from four datasets (Batista-Brito et al., 2008; Zhang et al., 2014; Zeisel et al., 2015, and the Gene Expression Nervous System Atlas (GENSAT) database) (Figure 1 and Supplementary Table S1). The FPKM (fragments per kilobase per million mapped reads) values corresponding to transcript levels in OPCs and astrocytes from postnatal day 7 (P7) mouse cortex were obtained from the RNA-seq database generated by Barres and colleagues (Zhang et al., 2014)^1^, from which we extracted FPKM values for the curated interneuron genes (Supplementary Table S2). Genes with FPKM <1 were considered “not expressed”. Gene Ontology analysis was performed with the web toolset g:Profiler (version r1750_e91_eg38) to identify enrichment of biological processes (Reimand et al., 2007, 2016) using the g:GOSt gene group functional profiling function. GO terms with *p*-value < 0.05 were considered significantly enriched.

RNA-seq data for interneuron genes expressed in normal adult skin tissue (dermal fibroblasts) were downloaded from accession GSE98157 as an FPKM transcript table (Zhao et al., 2019). Microarray expression data for interneuron genes expressed in mouse fibroblasts isolated from E13.5 embryos were downloaded from accession GSE8024 as processed data (Mikkelsen et al., 2007). RNA-seq data for mouse medial ganglionic eminence (MGE) isolated from E12.5 embryos were downloaded from accession GSE99049 as processed FPKMs (Liu et al., 2018).

### Chromatin-Immunoprecipitation Sequencing (ChIP-seq) Analysis

We obtained ChIP-seq data for genomic regions that were occupied by histone 3 lysine 27 acetylation (H3K27ac) and histone 3 lysine 4 tri-methylation (H3K4me3) in P2 rat cortical OPCs (Yu et al., 2013); histone 3 lysine 27 tri-methylation (H3K27me3) and H3K9me3 in P1 rat cortical OPCs, and histone 3 lysine 4 mono-methylation (H3K4me1) in mouse epiblast stem cell-derived OPCs (Najm et al., 2011). All animal experiments were approved by the Institutional Animal Use and Care Committees. The ChIP-seq data for H3K27ac and H3K4me3 were aligned to the rat rn5 genome build using Bowtie with the following options: -p 8 –best –chunkmbs 200^2^. Peak calling was performed using Model-based Analysis^3^ of ChIP-seq (MACS) with a *p* value cutoff of 1 × e^–9^. The ChIP-seq data of histone 3 lysine 27 tri-methylation (H3K27me3) and histone 3 lysine 9 tri-methylation (H3K9me3) were obtained from OPCs isolated from P1 rat cortices of either sex (Liu et al., 2015), and MACS was used for peak calling.

For analysis of histone marks in adult human astrocytes and adult dermal fibroblasts, H3K27ac, H3K4me3, H3K4me1, H3K27me3, and H3K9me3 ChIP-seq datasets were generated by the ENCODE Project Consortium (Consortium, 2012) and downloaded as narrowPeak files from the roadmap epigenomics project web portal^4^ and converted to BED files. ChIP-seq data for mouse astrocytes were obtained from embryonic stem (ES) cell-derived NPCs that were differentiated into mature astrocytes (Tiwari et al., 2018). EncodePeak files for H3K27ac and H3K4me1 ChIP-seq datasets were downloaded from the GEO database, accession GSM2535250, and converted to BED files. H3K27me3 ChIP-seq data for cortical astrocytes that were isolated at P5, expanded for 10 days and infected with EGFR-expressing viral supernatant were downloaded from the GEO database, accession GSE76289, as BED files (Signaroldi et al., 2016). For analysis of mouse adult dermal fibroblasts, H3K4me3 and H3K27me3 ChIP-seq datasets were downloaded from the GEO database, accession GSE58965, as BedGraph files and converted to BED files (Park et al., 2017). For analysis of histone marks in E13.5 mouse embryonic fibroblasts (MEFs), H3K27ac, H3K4me1, and H3K4me3, ChIP-seq datasets, broadPeak files were downloaded from the GEO database, accession GSE31039 generated by the mouse ENCODE project, and H3K27me3 and H3K9me3 wig files were downloaded from accession GSE26657 and converted to BED files. For analysis of histone marks of the MGE region of E12.5 telencephalon, H3K27ac, H3K4me1, H3K4me3, and H3K27me3 ChIP-seq datasets were downloaded from accession GSE85704 as MACS peak output files and converted to BED files (Sandberg et al., 2016).

All ChIP-seq BED files were used to call for closest genes using the closest feature utility in bedtools. ChIP peaks were parsed based on location, with ±2 kb from the gene transcription start site (TSS) defined as promoter, 2 kb downstream from gene TSS to 2kb downstream from the end of the last exon defined as gene body, and any peaks outside those regions defined as intergenic enhancer. Significant peaks were filtered with a false discovery rate ≤5% and *p*-value 1.00e^–05^. To compare RNA-seq expression and peak intensity of histone PTMs across different datasets, signal intensity values within each dataset in a given cell type were converted to percentiles, ranging from 100 for the gene with the highest mRNA expression or histone modification peak signal intensity to 0 for the gene with the lowest mRNA expression or peak signal intensity or no signal (Supplementary Table S3).

### Assay for Transposase Accessible Chromatin-Sequencing (ATAC-seq) Analysis

To assess chromatin accessibility, ATAC-seq data for open chromatin regions from P7 mouse cortical OPCs was downloaded from accession GSE116598 (Marie et al., 2018), and bigWig files were converted to BED files and BedGraph files. ATAC-seq data from adult mouse astrocytes infected with Xbp1-shRNA in an EAE model was downloaded from accession GSE121923 as BedGraph files and converted to BED files (Wheeler et al., 2019). Closest genes to ATAC-seq peaks were called as described above. For visualization of genome tracks, BedGraph files were uploaded to the Integrative Genomics Viewer (Thorvaldsdottir et al., 2013).

## Results

### Compiling Interneuron Genes

Since OPCs share an early developmental origin with cortical interneurons, we sought to determine whether OPCs had specific histone post-translational modifications (histone PTMs) at interneuron genes, which might facilitate their conversion into interneurons. We first curated a list of genes that are expressed specifically in interneurons at different stages of their development or known to be important for differentiation and maturation of interneurons from the following four sources (Figures 1A,B and Supplementary Table S1): (1) The top 500 genes that were enriched in acutely dissociated neurons from P8 cortex compared to genes expressed by OPCs, and this list included both excitatory and inhibitory neurons (Zhang et al., 2014); (2) 82 genes expressed in immature postmitotic interneuron precursors from E13.5 and E15.5 mouse neocortex (Batista-Brito et al., 2008); (3) 365 genes expressed in mature interneurons in young adult (P21-P31) somatosensory cortex and hippocampal CA1 region identified by single cell RNA-seq (Zeisel et al., 2015); and (4) 372 genes listed as interneuron-associated genes in the GENSAT database generated using text annotation search for “interneuron.” This resulted in a combined list of 890 non-duplicate genes (Figure 1B). We chose these four sources since they provided a diverse list of interneuron genes expressed at different developmental stages. This included genes that are important for the differentiation, function and subtype specification of interneurons (Batista-Brito et al., 2008; Rudy et al., 2011; Pla et al., 2018).

### Expression of Interneuron Genes in OPCs and Astrocytes

We previously noted from published transcriptomic analyses that OPCs express low levels of transcripts encoding some neuronal genes (Nishiyama et al., 2016). To systematically determine the levels of interneuron gene expression in OPCs, we generated a list of interneuron genes that had an FPKM >1 in the RNA-seq database generated from purified P7 mouse neocortical OPCs (Zhang et al., 2014). Of the 890 curated interneuron genes described above, 46% (405 genes) were expressed (FPKM > 1) in OPCs, with an average FPKM of 15.8 and median FPKM of 6.8 (Supplementary Table S2). Gene ontology analysis of interneuron genes expressed in OPCs revealed an enrichment of genes involved in nervous system development, neuronal projection development, and regulating membrane potential, ion transport and signal transduction (Figure 1C). Some of these enriched “interneuron” genes may play a role in OPC function such as process extension and regulation of membrane potential, supporting a shared function in OPCs and interneurons. We also examined interneuron gene expression in cortical astrocytes from P7 mice (Zhang et al., 2014) and found that compared to OPCs, fewer interneuron genes were expressed in astrocytes (330 genes, 37% of the 890 interneuron genes), with an average FPKM value of 13.2 and median FPKM of 5.4 (Supplementary Table S2). Gene ontology analysis of the interneuron genes expressed in astrocytes revealed that 6 of the 10 top GO terms were shared with those represented in OPCs. Unique functions for interneuron genes expressed in astrocytes included neurogenesis and regulation of synaptic transmission and neurotransmitter receptor activity, consistent with the known role of astrocytes at synapses (Figure 1D).

### Histone Post-translational Modifications (Histone PTMs) at Interneuron Genes in OPCs

We examined whether there were histone PTMs at a subset of interneuron genes in OPCs that could facilitate their reprograming into inhibitory neurons. Specifically, we were interested in determining whether interneuron genes in OPCs had an enrichment of bivalent histone PTMs. Bivalent genes are defined as genes that are occupied by both active and repressive histone PTMs. Many of the bivalently modified genes are developmentally important genes that regulate cell fate, and the bivalent marks are often resolved into either active or repressive marks as the cell differentiates into a more mature cell type, leading to transcriptional activation or silencing of the genes, respectively (Bernstein et al., 2006; Zhou et al., 2011). Thus, genes that are bivalently marked are considered to be repressed but “poised” for activation.

**FIGURE 2:**
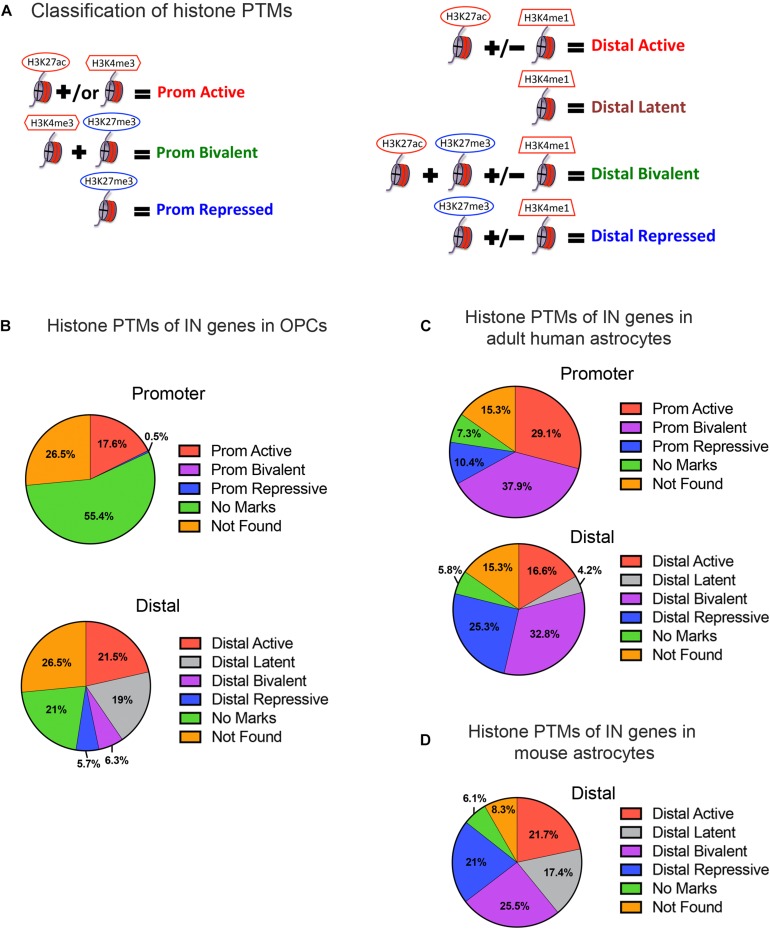
Histone post-translational modifications (PTMs) at interneuron genes in OPCs and astrocytes. **(A)** Diagram illustrating the operational classification of histone PTMs at the promoter and distal regions of interneuron genes used in this study. Red marks indicate active histone PTMs and blue marks indicate repressive histone PTMs. **(B)** The proportion of the 890 curated interneuron genes with each category of histone PTMs at promoter and distal regions in postnatal OPCs. **(C,D)** The proportion of interneuron genes with each category of histone PTMs at promoter and distal regions in adult human astrocytes **(C)**, and at the distal regions in mouse astrocytes derived from neural stem cells and matured in culture **(D)**.

To determine the key categories of histone PTMs associated with interneuron genes in OPCs, we analyzed ChIP-seq datasets from postnatal rodent OPCs for H3K4me1, H3K4me3, H3K27ac, and H3K27me3 (Yu et al., 2013; Liu et al., 2015; Factor and Tesar, unpublished) at the promoter and distal regions of the 890 interneuron genes in OPCs (Figure 2A). The promoter was defined as ±2kb from TSS (Roh et al., 2006), and distal region included gene body and intergenic regions. We used the following criteria to classify histone PTMs at interneuron genes. Promoter regions were classified into, (1) active histone PTM defined by H3K27ac occupancy with or without H3K4me3 (Barski et al., 2007; Creyghton et al., 2010; Sandberg et al., 2016); (2) bivalent histone PTM defined by the dual occupancy of the active mark H3K4me3 and the repressive mark H3K27me3 (Barski et al., 2007; Creyghton et al., 2010; Rada-Iglesias et al., 2011; Young et al., 2011; Zentner et al., 2011; Matsumura et al., 2015); and (3) repressive histone PTM defined by H3K27me3 occupancy without any of the above active marks (Bannister et al., 2001; Boyer et al., 2006; Barski et al., 2007; Zhu et al., 2013). Distal regions were classified into (1) active histone PTM defined by H3K27ac occupancy with or without H3K4me1 (Barski et al., 2007; Creyghton et al., 2010); (2) latent histone PTM defined by H3K4me1 occupancy alone (Barski et al., 2007; Guenther et al., 2007; Bogdanovic et al., 2012; Rada-Iglesias et al., 2012); (3) bivalent histone PTM defined by occupancy of H3K27ac alone or in combination with H3K4me1 and/or H3K27me3 (Zentner et al., 2011; King et al., 2016); and (4) repressive histone PTM defined by H3K27me3 occupancy alone or in combination with H3K4me1 (Attanasio et al., 2014). Genes marked with latent histone PTMs are considered to be ‘primed’ for activation, and this modification typically precedes H3K27ac deposition. Interneuron genes that lacked any of the above histone PTMs were grouped as “no marks,” and interneuron genes that were not found in the ChIP-seq data were classified as “not found.”

When we examined the histone PTMs at the 890 curated interneuron genes in postnatal mouse or rat OPCs, surprisingly none of the interneuron genes were bivalently marked at the promoter, and only 6.3% were bivalently marked at distal regions (Figure 2B). None of these were transcription factors known to be important for interneuron differentiation (Table 1). Among the interneuron genes, 17.5 and 21.5% had active histone modifications at promoter and distal regions, respectively (Figure 2B). While genes with H3K27ac had the highest transcript levels, those with H3K27ac positioned at both the promoter and gene body had higher transcript levels (average FPKM 32.87) than those with H3K27ac positioned at the promoter (average FPKM 24.42) or gene body (average FPKM 19.74) alone. Of the key interneuron transcription factor genes, Dlx2, Lhx6, and Sp9 were in this distal active category (Table 1).

**TABLE 1:** Histone post-translational modifications at key interneuron transcription factor genes in different cell types.

**OPC**			**Mouse astrocyte**		**Mouse adult fibroblast**	
**Gene**	**Promoter**	**Distal**	**Gene**	**Distal**	**Gene**	**Promoter**
Dlx1	No Marks	No Marks	Dlx1	No Marks	Dlx1	Bivalent
Dlx2	No Marks	Active	Dlx2	Active	Dlx2	Active
Dlx5	No Marks	No Marks	Dlx5	Repressive	Dlx5	Repressive
Dlx6	Not Found	No Marks	Dlx6	Repressive	Dlx6	Repressive
Lhx5	No Marks	Latent	Lhx5	Repressive	Lhx5	Repressive
Lhx6	No Marks	Active	Lhx6	Bivalent	Lhx6	Active
Lhx8	No Marks	Latent	Lhx8	Repressive	Lhx8	Repressive
Lhx9	No Marks	Latent	Lhx9	Repressive	Lhx9	Bivalent
sp8	No Marks	Latent	Sp8	Repressive	Sp8	Bivalent
sp9	No Marks	Active	Sp9	Bivalent	Sp9	Repressive

**MEF**			**MGE**			
**Gene**	**Promoter**	**Distal**	**Gene**	**Promoter**	**Distal**	

Dlx1	Active	Latent	Dlx1	Bivalent	Bivalent	
Dlx2	Active	Bivalent	Dlx2	Bivalent	Bivalent	
Dlx5	Bivalent	Repressive	Dlx5	Bivalent	Bivalent	
Dlx6	Bivalent	Repressive	Dlx6	Bivalent	Bivalent	
Lhx5	Active	Repressive	Lhx5	Bivalent	Bivalent	
Lhx6	Active	Active	Lhx6	Bivalent	Bivalent	
Lhx8	Active	Active	Lhx8	Bivalent	Bivalent	
Lhx9	Active	Bivalent	Lhx9	Bivalent	Bivalent	
Sp8	Active	Latent	Sp8	Bivalent	Bivalent	
Sp9	Active	Repressive	Sp9	Bivalent	Bivalent	

**Human Astrocyte**			**Human Adult Fibroblast**			
**Gene**	**Promoter**	**Distal**	**Gene**	**Promoter**	**Distal**	

Dlx1	Bivalent	Repressive	Dlx1	Bivalent	Repressive	
Dlx2	Bivalent	Bivalent	Dlx2	Active	Active	
Dlx5	Bivalent	Repressive	Dlx5	Bivalent	Repressive	
Dlx6	Bivalent	Repressive	Dlx6	Bivalent	Repressive	
Lhx5	Repressive	Repressive	Lhx5	Repressive	Repressive	
Lhx6	Repressive	Bivalent	Lhx6	Repressive	Repressive	
Lhx8	Repressive	Repressive	Lhx8	Bivalent	Repressive	
Lhx9	Repressive	Bivalent	Lhx9	Bivalent	Repressive	
Sp8	Bivalent	Repressive	Sp8	Bivalent	Repressive	
Sp9	Bivalent	Repressive	Sp9	Active	Active	

*Red, active marks; orange, latent mark; green, bivalent marks; blue, repressive marks; gray, no marks; purple, gene not found in the ChIP-seq dataset.*

Only 0.5% of the interneuron gene promoters and 5.7% of distal regions were repressively marked in OPCs. The majority of interneuron genes (55.4%) lacked any of the analyzed histone PTMs at the promoter, and 21% of the genes also lacked the analyzed PTMs at distal regions. This group of interneuron genes in OPCs with “no marks” at the promoter included all but one of the ten key interneuron transcription factor genes (Dlx1, Dlx2, Dlx5, Lhx6, Lhx8, Lhx9, Sp8, and Sp9). Dlx1, Dlx5, and Dlx6 also lacked the analyzed histone PTMs at distal sites (Table 1). One-fifth of interneuron genes were latently marked at distal regions, including transcription factors Lhx5, Lhx8, Lhx9 and Sp8, suggesting these genes were in a chromatin state “primed for activation.”

### Histone PTMs at Interneuron Genes in Astrocytes

We next examined whether the number and extent of histone PTMs at interneuron genes differed between OPCs and astrocytes, which represent another non-neuronal neuroectodermally derived cell type. We first compared human adult astrocytes with OPCs because ChIP-seq data for all four histone PTMs at the promoter were not available for mouse astrocytes. The most striking difference between mouse OPCs and human astrocytes was the abundance of bivalent histone PTMs at the 890 interneuron genes in astrocytes both at promoter and distal regions, which represented one-third of the interneuron genes (Figure 2C), compared to that in OPCs. A significantly larger proportion of the interneuron genes had repressive marks in astrocytes than in OPCs at the promoter or distal sites. All the key interneuron transcription factor genes had either bivalent or repressive marks in astrocytes (Table 1). Two other major differences between OPCs and human astrocytes were the larger proportion of interneuron genes in OPCs with no marks at the promoter or distal sites and those with distal latent marks compared with astrocytes. More interneuron genes in astrocytes had active marks at the promoter than those in OPCs, while at distal regions, the proportion of actively marked interneuron genes was slightly lower in astrocytes than in OPCs.

To determine whether the observed differences in histone PTMs at interneuron genes between murine OPCs and human astrocytes were due to species differences or a reflection of the differences between the cell types, we performed a similar analysis on mouse cells. Since a comparable ENCODE ChIP-seq datasets from acutely isolated mouse astrocytes was not available, we used H3K27ac and H3K4me1 ChIP-seq datasets from ES cell-derived NPCs that had been further differentiated into astrocytes (Tiwari et al., 2018) and H3K27me3 ChIP-seq dataset from astrocytes isolated from P5 mouse cortex and expanded for 10 days in culture (Signaroldi et al., 2016). These astrocytes exhibited some phenotype of mature astrocytes, such as the expression of Aquaporin-4 and genes involved in signaling and cytokine response (Tiwari et al., 2018). Since H3K4me3 ChIP-seq data was unavailable for mouse astrocytes, we analyzed distal regions only. The abundance of bivalently and repressively marked genes was similar in human and mouse astrocytes and much greater than that in OPCs (Figures 2B–D). A notable difference between mouse and human astrocytes was the higher proportion of interneuron genes that were latently marked (Figure 2D), which was comparable to that in OPCs and could reflect the degree of cell maturity rather than species difference. The proportions of actively marked interneuron genes in mouse astrocytes was slightly higher compared to human astrocytes and comparable to that in OPCs. The key interneuron transcription factor gene Dlx2 had a distal active mark in both OPCs and mouse astrocytes, while the other two transcription factor genes Lhx6 and Sp9 that had active distal PTMs in OPCs had distal bivalent marks in mouse astrocytes, and those with latent marks in OPCs (Lhx5, 8, 9, and Sp8) had distal repressive marks in mouse astrocytes. The proportion of interneuron genes with no marks was similar between human and mouse astrocytes and represented a significantly lower fraction than those with no marks in OPCs. Overall, the distribution of histone PTMs at interneuron genes was highly conserved between the mouse and human, and the most significant differences in histone PTMs at interneuron genes between OPCs and astrocytes were the higher occupancy of bivalent and repressive marks in astrocytes and the paucity of genes with no marks.

### Histone PTMs at Interneuron Genes in Fibroblasts

**FIGURE 3:**
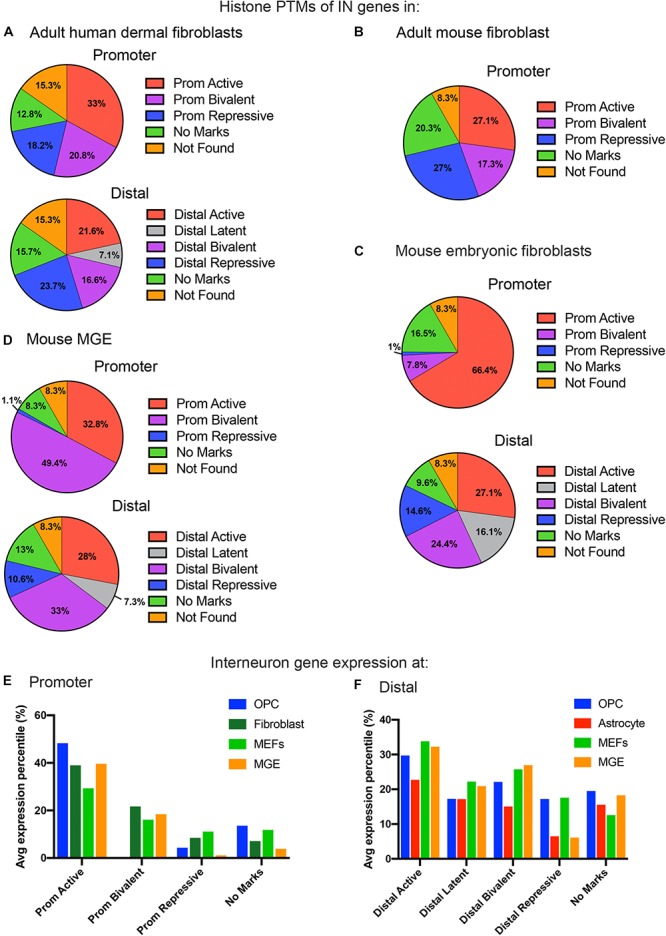
Histone PTMs at interneuron genes in embryonic and adult fibroblasts and MGE cells. Distribution of histone PTMs at promoter and distal regions of all 890 curated interneuron genes in adult human fibroblasts **(A)**, mouse fibroblasts **(B)**, mouse embryonic fibroblasts **(C)**, and mouse MGE **(D)**. Bar graph of average expression percentiles (*y*-axis) of interneuron genes expressed in OPCs (blue), astrocytes (red), adult fibroblasts (dark green), and embryonic fibroblasts (light green) for each histone PTM category (*x*-axis) at the promoter **(E)** and distal regions **(F)** of interneuron genes.

We next compared histone PTMs at interneuron genes between OPCs and fibroblasts. Fibroblasts are mesodermally derived and are often targeted for direct reprograming. We reasoned that the mesodermal origin of fibroblasts would result in a more closed chromatin environment around interneuron genes, with greater repressive and lower bivalent or active marks. We first examined adult human dermal fibroblasts, for which ChIP-seq data for all the histone PTMs were available. The proportion of repressive and bivalent marks at interneuron genes was significantly higher both at the promoter and distal regions in adult human fibroblasts compared to murine OPCs (Figure 3A). The proportion of these marks was highly conserved in adult (8-week-old) mouse dermal fibroblasts (Figures 3A,B). Surprisingly, about one-third of the interneuron genes in mouse and human fibroblasts had active marks at the promoter, similar to astrocytes and higher than that in OPCs. Fibroblasts had a similar proportion of latently marked genes to astrocytes, which was lower than that in OPCs. The interneuron transcription factor Dlx2 had active promoter marks in both mouse and human dermal fibroblasts, while the other interneuron transcription factor genes had either repressive or bivalent marks in fibroblasts, with the exception of active promoter marks on Lhx6 in mouse fibroblasts and active promoter marks on Sp9 in human fibroblasts (Table 1).

In addition to adult fibroblasts, we examined histone PTMs at interneuron genes in fibroblasts isolated from E13.5 mouse embryos to explore age-dependent differences (Figure 3C). Mouse embryonic fibroblasts (MEFs) are a population of immature fibroblasts that have been widely used as a starting population for reprograming. The most notable characteristic of histone PTMs in MEFs was that two-thirds of interneuron genes had active modifications at the promoter, which was significantly higher than that in any other cell types examined, including OPCs. This was accompanied by a lower proportion of bivalent and repressive marks at the promoter compared to adult fibroblasts, though they were higher than in OPCs. The distal histone PTMs in MEFs were similar to those in adult fibroblasts except for the larger proportion of latently marked genes in MEFs, which was comparable to that in OPCs. In MEFs, the key interneuron transcription factor genes Dlx1, Dlx2, Lhx5, Lhx6, Lhx8, Lhx9, Sp8 and Sp9 had active modifications at the promoter, while Dlx5 and Dlx6 were bivalently modified at the promoter (Table 1). These findings suggest that cells from developmentally immature animals tended to have more active promoter and latent distal histone PTMs and fewer genes with repressive marks than those from more mature animals.

### Histone PTMs in Cells From Mouse Medial Ganglionic Eminence (MGE)

We examined histone PTMs at interneuron genes in cells from E12.5 MGE as an example of progenitors that were fated to become inhibitory neurons. The MGE at this developmental age consists of neural progenitors, neuroblasts, post-mitotic differentiating inhibitory neurons and a small population of progenitor cells that are becoming committed to the oligodendrocyte lineage. The most striking feature of the histone PTMs in MGE cells was the abundance of interneuron genes with bivalent marks, particularly at the promoter, which comprised almost half of the interneuron genes (Figure 3D) and was higher than in any other cell types, consistent with the presence of multipotent progenitors in this region. Notably, all the key interneuron transcription factor genes had bivalent marks at the promoter and distal sites (Table 1). MGE cells also had a higher proportion of interneuron genes with active marks at the promoter and distal regions compared with OPCs, though this was lower than that in MEFs. The proportion of repressive modification at the promoter region of interneuron genes was similar to that in MEFs and OPCs and slightly higher than in OPCs at distal regions. These findings suggest that interneuron genes were more highly decorated with bivalent histone PTMs in MGE cells than in other cell types, consistent with previous reports on bivalent marks in uncommitted progenitor cells (Bernstein et al., 2006; Boyer et al., 2006; Mikkelsen et al., 2007).

### Expression of Interneuron Genes in Mouse OPCs, Astrocytes, Fibroblasts, and MGE Cells

To determine if the histone PTM occupancy at interneuron genes were correlated with transcription, we compared interneuron genes in each histone PTM category to the RNA-seq data of different cell types. The FPKM values of interneuron genes in P7 cortical OPCs and astrocytes (Zhang et al., 2014) and the histone PTM patterns for OPCs are shown in Supplementary Table S2. We extended the analysis of interneuron gene expression levels with histone PTM occupancy across the different mouse cell types. Since the different methods of transcriptome analyses of the various mouse cell types precluded a direct comparison of FPKM values, we normalized the range of FPKM or microarray expression values within each RNA-seq or microarray dataset to obtain percentiles of transcript expression for each cell type, ranging from highest at 100th percentile to lowest at 0th percentile, and compared the percentile values for interneuron genes among OPCs, mouse astrocytes, adult mouse fibroblasts, MEFs, and MGE.

Overall, the levels of interneuron transcripts with each histone PTM category tended to be higher in OPCs than in astrocytes, with the exception of latently marked interneuron genes, which were expressed at comparable levels in OPCs and astrocytes (Figures 3E,F). When comparing transcript levels of interneuron genes marked by the different histone PTMs, interneuron genes with active modifications at either the promoter or distal regions had the highest average expression percentile in all cell types, as expected. In OPCs, 83% of the interneuron genes with active marks at the promoter had FPKM values above 1, and the majority of interneuron genes with FPKM values above 100 had an active modification at the promoter and/or distal region (Supplementary Table S2).

Generally, there was a good correlation between histone PTMs at the promoter and transcript levels (Figure 3E). Those with active marks had the highest level of expression, those with repressive marks had the lowest expression, and those with bivalent marks had intermediate levels of expression. Interneuron genes with no marks at the promoter had a wide range of expression, from <1 to >100 FPKM, but the average expression levels of these genes were significantly lower than those with active marks, and this was true for all cell types. The average expression level of interneuron genes with no marks at the promoter was higher than those with repressive marks. However, in OPCs, the nine key interneuron transcription factor genes described above that had no marks at the promoter all had FPKM values of <1, which is consistent with the non-neuronal phenotype of OPCs.

In OPCs, the expression levels of interneuron genes with different types of histone PTMs at distal sites did not segregate as cleanly as the promoter marks. While the genes with distal active marks had the highest levels of expression, those with latent, bivalent, repressive or no marks at distal sites were expressed at similar levels in OPCs. By contrast, in astrocytes, there was a tighter correlation between expression levels and distal histone PTMs, similar to the histone PTMs at the promoter. Thus, interneuron genes with bivalent or repressive marks were more repressed in astrocytes than in OPCs.

### Quantitative Analysis of Histone PTMs at Interneuron Genes With Bivalent and Repressive Marks

We explored further into the nature of the bivalent modification that was detected at an unexpectedly large number of interneuron genes in astrocytes and fibroblasts from both human and mouse. The analysis described above did not take into account the ChIP-seq peak signal intensity of each kind of histone PTM. To more quantitatively examine the histone PTMs at interneuron genes, we normalized the range of signal intensities within a histone PTM dataset to obtain percentiles of signal intensities, ranging from highest at 100th percentile to lowest at 0th percentile, and compared the percentile values for each type of histone PTM at the interneuron genes among murine OPCs, human adult astrocytes, mouse astrocytes, and human adult dermal fibroblasts. We were unable to include the MEFs in this comparison because there was no quantitative output from the available ChIP-seq data. We limited our analysis to distal sites because there were too few interneuron genes with bivalent or repressive marks at the promoter region in OPCs for a meaningful comparison.

**FIGURE 4:**
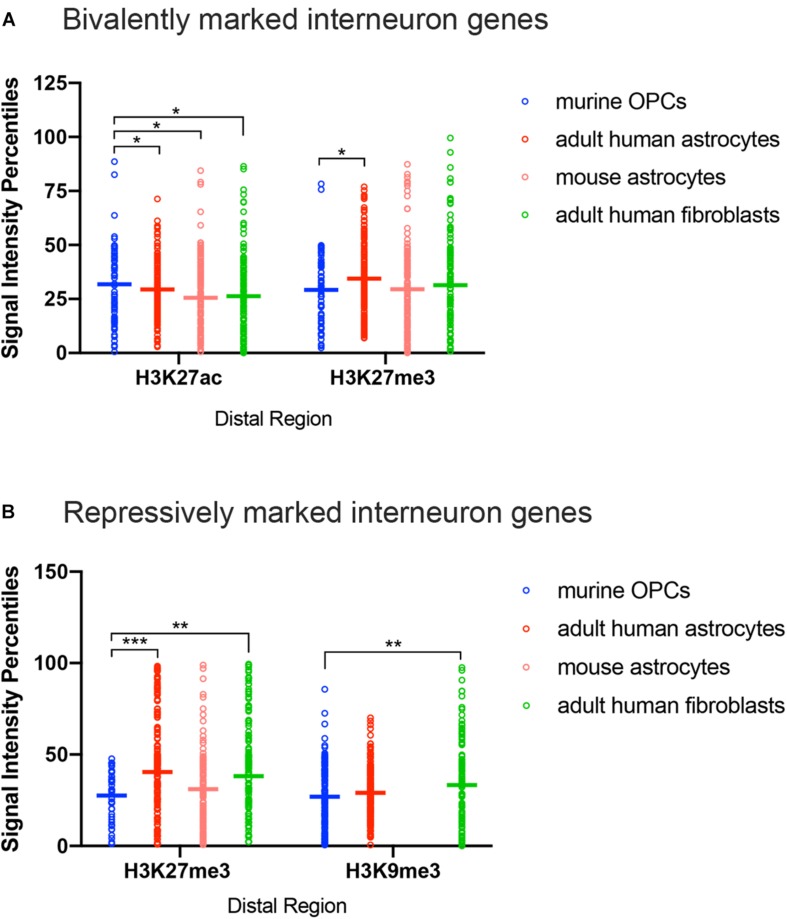
ChIP-seq peak signal intensity at interneuron genes in OPCs and other cell types. Dot plots show signal intensity percentiles (*y*-axis) for histone PTMs (*x-*axis) at interneuron genes in OPCs (blue), human astrocytes (red), mouse astrocytes (pink), and adult human fibroblasts (light green) among the bivalently marked **(A)** and repressively marked **(B)** interneuron genes. Each circle represents the signal intensity percentile data after binning of two adjacently ranked genes. Horizontal bars represent the means of the signal intensity percentiles within each histone PTM dataset. ^*^p < 0.05, ^∗∗^*p* < 0.01, ^∗∗∗^*p* < 0.001, two-way ANOVA, Fisher’s LSD.

The most notable difference between OPCs and astrocytes or adult dermal fibroblasts was that the signal intensity of H3K27me3 at distal regions of interneuron genes with both bivalent and purely repressive histone PTMs was significantly lower in OPCs compared to human and mouse astrocytes and adult human fibroblasts (Figures 4A,B). When comparing the range of the occupancy of the H3K27me3 mark at distal sites in both bivalently and repressively marked interneuron genes, the majority of the genes with H3K27me3 in OPCs had signal intensity values of less than 50th percentile, whereas some of the highest degrees of enrichment for H3K27me3 at interneuron genes were found in astrocytes and fibroblasts. We included an analysis of H3K9me3 as another repressive modification and an indicator of heterochromatin. The difference between the depth of H3K9me3 enrichment at interneuron genes in OPCs and astrocytes was not as prominent as the difference in H3K27me3, while fibroblasts had a significantly greater deposition of H3K9me3 compared to OPCs. These observations indicated a tendency for many of the interneuron genes to be more heavily enriched for the repressive histone PTM H3K27me3 in astrocytes and fibroblasts than in OPCs, consistent with lower levels of interneuron transcripts in astrocytes compared with OPCs.

### Chromatin Accessibility in Murine OPCs and Mouse Astrocytes

**FIGURE 5:**
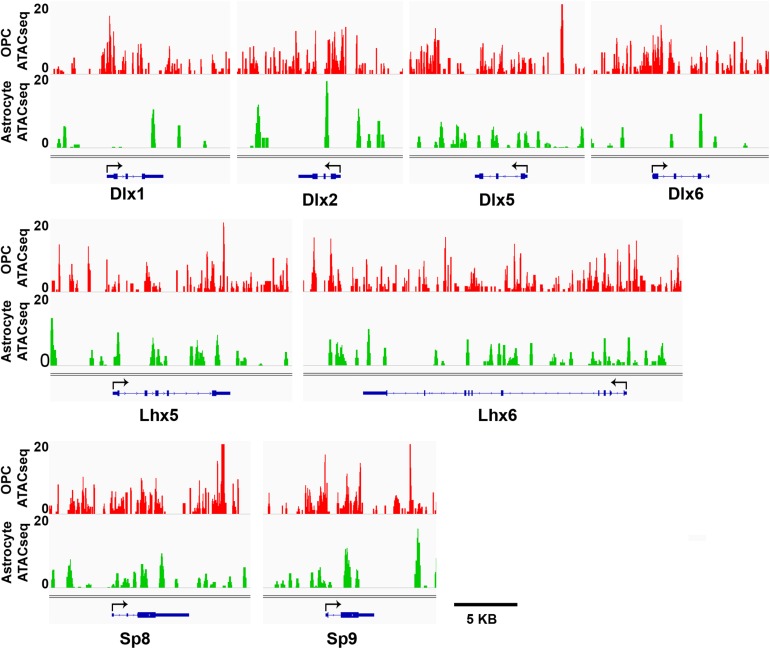
ATAC-seq genome tracks showing open or closed chromatin regions at key interneuron transcription factor genes. Tracks show peak signal intensity (*y-*axis) for open chromatin regions of interneuron genes (*x*-axis) in mouse OPCs (red, **top**) and mouse astrocytes (green, **bottom**). Individual gene maps are shown in blue. Tracks span +5 kb from first exon and –5 kb from last exon of each gene. Black arrows indicate transcription start site and direction of transcription. Scale bar = 5 kb.

We have shown that OPCs have the highest proportion of interneuron genes that lacked the four histone modifications analyzed, particularly at key interneuron transcription factors (Table 1), while this group of “unmarked” genes had the highest expression of interneuron genes among all mouse cell types analyzed (Figures 3E,F). In addition to histone PTMs, the chromatin structure also critically affects transcription by modulating accessibility of transcription factors (Thurman et al., 2012). ATAC-seq is a method that uses a mutant Tn5 transposase to interrogate across the genome for accessible and hence open chromatin, which can be quantified by degree of transposase-mediated insertion of sequencing adaptors, measured by the number of sequencing reads. We analyzed available ATAC-seq datasets from P7 mouse cortical OPCs (Marie et al., 2018) and adult mouse cortical astrocytes (Wheeler et al., 2019) to examine chromatin accessibility around the key interneuron transcription factors. OPCs had sizeable open chromatin peaks around the TSS and the first exons of Dlx1, Dlx2 and Dlx6 genes that were largely absent or very sporadic in astrocytes (Figure 5). This is consistent with the previous report that genes that are transcribed typically have a large chromatin peak at the TSS as well as the transcription termination site (Teif et al., 2012), and suggests a more transcription-conducive environment at these genes in OPCs. Other key interneuron transcription factor genes that were unmarked in OPCs, including Dlx5, Lhx5, Lhx6, and Sp8 also had significant ATAC-seq reads in OPCs but the peaks appeared similar between OPCs and astrocytes and seemed more randomly distributed throughout the genes. Both OPCs and astrocytes had a chromatin peak around the first exon of the Sp9 gene, but the intensity was much greater in OPCs. This supports the notion that interneuron genes lacking the four histone modifications have a permissive environment and thus may be “poised” for transcription.

## Discussion

Gene expression is globally regulated by transcription factor availability and the chromatin environment. Histone PTMs and ATP-dependent chromatin remodeling complexes play key roles in defining the chromatin landscape of a given cell type under different conditions. We focused this study on examining histone PTMs at interneuron genes in OPCs as a first step toward gaining a mechanistic insight into how OPCs can be reprogramed toward an interneuron fate. We were particularly interested in determining whether histone PTMs at interneuron genes in OPCs were distributed in a way that marked them in a “poised state”. Bivalent histone modifications are characterized by the presence of both active and repressive histone PTMs. During development, the bivalent marks are often resolved into either active or repressive marks as the cell differentiates from a progenitor state into a mature cell type, resulting in transcriptional activation or silencing of the genes, respectively (Bernstein et al., 2006; Zhou et al., 2011). Since OPCs that arise from ventral sources, which comprise about half of those in the neocortex and are lineally closely related to interneurons, our initial hypothesis was that OPCs have an enrichment of bivalent histone PTMs at interneuron genes, making them “poised for activation,” compared to other cell types such as astrocytes and fibroblasts, which are developmentally more distant from interneurons. However, contrary to our expectations, we found that bivalent modifications were the least abundant in OPCs at interneuron genes, and a large majority of interneuron genes either had active or no histone PTMs at their promoter, while bivalent marks were a prominent feature of the promoter of interneuron genes in the MGE.

### Active and Latent Histone PTMs at Interneuron Genes

Using the available transcriptomic data, we found that interneuron genes were expressed at a higher level in OPCs than in astrocytes. This led us to closely examine active histone PTMs in these cells. H3K27ac is a well-characterized active histone mark correlated with enhancer activity (Barski et al., 2007), and H3K4me3 has traditionally been associated with active promoters, although it is also deposited at 59% of silent promoters (Barski et al., 2007; Guenther et al., 2007). H3K4me1 is associated with active enhancers but also functions as a ‘priming’ mark, identifying genes that will become active (Creyghton et al., 2010; Rada-Iglesias et al., 2011). For this reason, we classified promoters with H3K4me3 and/or H3K27ac and distal regions of genes with H3K27ac with or without H3K4me1 as active. Distal regions associated with H3K4me1 without the other active PTMs were classified as latent. The presence of H3K27ac at both the promoter and gene body showed the greatest correlation with higher transcript levels, as was the presence of active promoter PTMs, consistent with previous reports. The proportion of interneuron genes with active histone PTMs was unexpectedly high in fibroblasts, as we had predicted that interneuron genes would be more permanently repressed in mesodermally derived cells. It is possible that histone marks do not affect chromatin structure by themselves but influence the binding or activity of other chromatin regulators, such as ATP-dependent chromatin remodeling enzymes (Zentner and Henikoff, 2013). The abundance of genes with active promoter marks in fibroblasts was even greater in MEFs, which could reflect the generally high degree of open chromatin and active transcriptional state in embryos.

In OPCs, there were more interneuron genes with latent marks than in other cell types, particularly those from adults, suggesting that this could be a PTM that has a more significant function in cells during development. Consistent with the “priming” function known for the H3K4me1 mark, the transcript levels of these genes were comparable to those of distal bivalently marked genes and lower than those of actively marked genes.

### Bivalent and Repressive Histone PTMs at Interneuron Genes

Contrary to our prediction that many of the interneuron genes have bivalent marks in OPCs, none of the interneuron genes had bivalent marks at the promoter in OPCs, and bivalent marks were more frequently detected in all the other cell types. Moreover, the relative abundance of bivalent marks was highly conserved in human and mouse astrocytes. When we examined quantitatively the degree of enrichment of each of the specific histone PTMs classified as bivalent marks, the repressive H3K27me3 mark was significantly more enriched at interneuron genes in adult human astrocytes than in OPCs. This was not the case with mouse astrocytes, which had been cultured from NPCs and matured *in vitro* for 5 days. Thus, the higher H3K27me3 deposition at bivalently marked genes in astrocytes could reflect age-dependent differences, rather than a species difference, and it is possible that OPCs from the adult brain have a greater enrichment of H3K27me3 at interneuron genes. While H3K27me3 or Ezh2, the Polycomb group methyltransferase that catalyzes the deposition of this PTM, has been detected at some interneuron genes (Sher et al., 2012; Liu et al., 2015), our analyses revealed a greater occupancy of the active H3K27ac mark at distal bivalently modified interneuron genes in OPCs compared to other cell types. Collectively, these observations indicate that the interneuron genes were less repressed in OPCs, consistent with the higher average FPKM of bivalently marked interneuron genes in OPCs than in astrocytes. Similarly, among the repressively marked genes, there was a greater enrichment of H3K27me3 in adult astrocytes and fibroblasts than in OPCs. In contrast to H3K27me3, H3K9me3 occupancy at interneuron genes in OPCs was similar to that in astrocytes but lower than that in fibroblasts. It is possible that this modification plays a more important role in permanently repressing interneuron genes in non-neurectodermally derived cells. Compared to OPCs, H3K9me3 has been shown to be more abundant in mature oligodendrocytes, and many of the genes occupied by H3K9me3 in oligodendrocytes are genes related to GABAergic transmission and neuronal differentiation (Liu et al., 2015). Thus, it appears that in the terminally differentiated oligodendrocytes, H3K9me3-mediated repression of interneuron genes occurs more prominently than in astrocytes and that the interneuron fate is more tightly sealed. This is consistent with the observation that astrocytes can be reprogramed into interneurons under certain conditions (Heinrich et al., 2010; Niu et al., 2013).

### No Histone PTMs at Many Interneuron Genes in OPCs, Particularly the Key Transcription Factor Genes

A major unexpected observation was the large number of interneuron genes in OPCs that had none of the four histone marks at either their promoter or distal regions. It was intriguing that all but one of the ten key interneuron transcription factors in OPCs had no marks at their promoter (Table 1). Several observations make it highly unlikely that this group arose from technical reasons such as inadequate peak detection of the ChIP-seq data. First, other modifications, such as active marks were detected at the promoters in OPCs. Second, 7 out of 10 of these transcription factor genes had latent or distal active marks that were distinct from those in other cell types. Third, ATAC-seq data revealed greater chromatin accessibility around the transcription initiation sites of these genes in OPCs compared to that in astrocytes in which these genes were more prominently marked by bivalent and repressive marks. Collectively, these observations indicate that interneuron genes with no promoter marks represent a specific functional state that can be considered as a ‘poised’ state, somewhat similar to bivalently or latently marked genes. It is possible that they represent a transition from an active to a more repressed state or the converse as the cells develop further along the oligodendrocyte lineage, analogous to the bivalent marks in multipotent stem cells. Notably, all ten key interneuron transcription factor genes were bivalently marked in E12.5 MGE cells, which is consistent with the original description of bivalent histone PTMs prior to lineage restriction from multipotent stem cells, which are resolved to either active or repressive marks upon lineage commitment (Bernstein et al., 2006; Boyer et al., 2006). It would be interesting to examine the evolution of the PTMs at these genes throughout different stages of oligodendrocyte development.

The OPCs that were used for ChIP-seq analyses in this study were mostly derived from neocortical OPCs from perinatal rodents, which represent a mixture of OPCs derived from ventral germinal zones and those from the dorsal Emx1 domain (Kessaris et al., 2006; Winkler et al., 2018). The paucity of repressive histone PTMs at interneuron genes in OPCs could reflect a unique property of ventrally derived OPCs that share their origin with interneurons, and that this signal is diluted by dorsally derived OPCs with a different histone PTM signature. Conversely, interneuron genes in OPCs from the ventral sources might require tighter repression when their fate diverges from a common precursor to firmly establish their oligodendrocyte lineage identity, and that the paucity of repressive marks in cortical OPCs reflects the property of dorsally derived OPCs diluted by ventrally derived OPCs with a different PTM signature. It is also possible that the histone modifications do not reflect the origin of OPCs but rather the function of OPCs and the necessity to transcribe some genes expressed in interneurons to maintain OPC functions. Comparison of ChIP-seq data of OPCs from ventral and dorsal germinal zones should provide a clearer answer as to whether the developmental origin and relation to interneurons plays a significant role in their chromatin landscape. Regardless of the possible heterogeneity among OPCs in their histone modifications, the lack of repressive histone PTMs and the open chromatin state at these key interneuron transcription factor genes found in the neocortical OPCs could give OPCs a significant advantage over other cell types for reprograming into interneurons.

### Species-Dependent Differences in Histone PTMs

We initially compared histone modifications of murine OPCs and human astrocytes and fibroblasts, which was supplemented with data from mouse astrocytes and fibroblasts where available. Although a comprehensive analysis of all four histone modifications of the different cell types done within the same species would have been ideal to fully validate the findings of this study, such a study was not feasible with the currently available datasets, and our findings suggested a high degree of species conservation of histone PTMs at interneuron genes. For example, the proportional distribution of the histone modifications was similar between mouse and human astrocytes, with the exception of more distal latent modified genes in mouse astrocytes. Furthermore, there was a higher proportion of genes that were bivalently and repressively marked in both mouse and human astrocytes compared to OPCs, also suggesting species conservation. A similar conservation was observed for fibroblasts, which showed similar extent of active marks at the promoter in mouse and human. Regardless of the species, more interneuron genes were repressively marked and bivalently marked in fibroblasts than in OPCs. Consistent with our findings, a study on the direct comparison of histone PTMs between mouse and human brain tissue showed 90% conservation at promoter regions, 84% at enhancers, and 33% of heterochromatin regions (Gjoneska et al., 2015). A separate group performed a similar study and found a strong association between stability and conservation of histone modifications in mouse and human species (Woo and Li, 2012). Thus, the observed differences seen among histone PTMs at interneuron genes between mouse and human cells are more likely due to cell type-dependent and age-dependent differences than inherent interspecies differences.

In summary, we have identified a characteristic histone PTM signature at interneuron genes in OPCs, which consisted of an enrichment of active histone PTMs and a paucity of bivalent and repressive modifications, particularly H3K27me3, compared with adult astrocytes and fibroblasts. In both OPCs and astrocytes, the histone PTM signature was highly correlated with transcript levels. MEFs, on the other hand, had a greater enrichment of active histone PTMs at their interneuron genes, suggesting that age significantly influences the chromatin landscape. Most somatic cell reprograming strategies require the bHLH transcription factor Ascl1 (Wapinski et al., 2013), which is considered a pioneer transcription factor capable of opening nucleosome-bound chromatin (Zaret and Carroll, 2011; Wapinski et al., 2017). Our findings that OPCs had a more accessible chromatin environment around their key interneuron transcription factor genes and lacked repressive marks could be partially explained by the expression of Ascl1 in OPCs (Nakatani et al., 2013; Zhang et al., 2014), suggesting that OPCs could more readily switch their fate into interneurons than adult fibroblasts or astrocytes, given the correct signals. The observation that OPCs in the ventral telencephalon (striatum) were readily reprogramed into interneurons (Torper et al., 2015; Pereira et al., 2017), while similar attempts in the neocortex, were only successful in the injured environment (Guo et al., 2014; Heinrich et al., 2014), could be related to differences in their epigenetic landscape around interneuron genes that could reflect their developmental origin. Further explorations on age-, cell type-, and cell origin-dependent differences in the chromatin landscape could lead to rational approaches for manipulating the fate of OPCs and exploiting the lineage plasticity of this ubiquitous and abundant self-renewing cell population.

## Statement of Data Sharing

We have provided the key datasets as Supplementary Tables. Additional datasets that we have generated will be shared upon request.

## Ethics Statement

The study was approved by the Institutional Animal Care and Usage Committee.

## Author Contributions

LB and AN conceived the study and wrote the manuscript. DF, JL, CZ, QL, PC, and PT provided the ChIP-seq data. VS extracted the ChIP-seq data. LB analyzed the data with the help of VS and IM.

## Conflict of Interest Statement

The authors declare that the research was conducted in the absence of any commercial or financial relationships that could be construed as a potential conflict of interest.

## Footnotes

1 https://web.stanford.edu/group/barres_lab/brain_rnaseq.html

2 http://bowtie-bio.sourceforge.net

3 http://liulab.dfci.harvard.edu/MACS

4 https://egg2.wustl.edu/roadmap/web_portal/

## Supplementary Material

TABLE S1 List of interneuron genes obtained from the four sources.

TABLE S2 Expression levels of the interneuron genes expressed in OPCs and astrocytes and histone PTMs in OPCs. Histone PTM categories are color coded as follows: red, active marks; orange, latent mark: blue, repressive marks; green, bivalent marks; gray, no marks detected; and purple, genes not found in the ChIP-seq datasets. Specific histone PTMs are indicated in red font for active modifications and blue font for repressive modifications.

TABLE S3 Signal intensities of the ChIP-seq peaks for each of the histone modifications at the 890 curated interneuron genes in different cell types. Signal intensity values within a histone PTM dataset were obtained by converting the ChIP peaks to percentiles, ranging from 100 (highest signal intensity) to 0 (no signal). The datasets for OPCs, astrocytes and fibroblasts are in separate worksheets.
